# Socio-Economic Factors Influencing Intimate Partner Violence Among Adolescents and Young Women in Sub-Saharan Africa: A Scoping Review

**DOI:** 10.3389/phrs.2024.1607041

**Published:** 2025-01-03

**Authors:** Olutoyin Opeyemi Ikuteyijo, Nejimu Zepro, Akanni Ibukun Akinyemi, Nicole Probst-Hensch, Sonja Merten

**Affiliations:** ^1^ Department of Public Health and Epidemiology, Swiss Tropical and Public Health Institute, University of Basel, Basel, Switzerland; ^2^ Faculty of Medicine, University of Basel, Basel, Switzerland; ^3^ College of Medical and Health Science, Samara University, Semera, Ethiopia; ^4^ Department of Demography and Social Statistics, Obafemi Awolowo University, Ile Ife, Osun, Nigeria

**Keywords:** socioeconomic status, adolescent health, sub-Saharan Africa, intimate partner violence, adolescent girls, young women

## Abstract

**Objective:**

To summarize the evidence on how socio-economic status and intimate partner violence (IPV) are interrelated among adolescents and young women (AYW) in sub-Saharan Africa (SSA).

**Methods:**

Online databases such as MEDLINE, Embase, PsycIFO, CINAHL, Cochrane Central, Sociological Abstracts, Web of Science, and the African Index Medicus were used to identify studies published between 2015 and 2022. The reporting procedure was the Preferred Reporting Item for Systematic Reviews and Meta-Analysis (PRISMA) as a checklist extension for the scoping review.

**Results:**

The majority of the publications, ten (62.5%) were cross-sectional studies, while four (25%) were qualitative studies and two (12.5%) were mixed methods studies. The review found that lack of financial resources exposed AYW to all forms of violence ranging from physical, sexual, emotional, psychological, and economic violence. Nonetheless, financial reliance on a partner poses a long-term threat to AYW employment and financial stability.

**Conclusion:**

Socio-economic status influences the level of IPV experienced by AYW in many countries of SSA, while evidence of the long-term effects remains uncovered. To achieve Sustainable Development Goal (SDG) 1, which focuses on ending poverty in all forms by 2030, socio-economic inequalities caused by IPV among AYW require prompt interventions.

## Introduction

Violence is the second leading cause of death among adolescent girls and young women aged 15–24 years [1], a population of nearly 880 million, representing 12% of the world population [2]. The World Health Organization (WHO) estimates that 29.4% of adolescent girls worldwide have experienced intimate partner violence (IPV) during their lifetime [3]. Globally, 85% of violence perpetrations [4] and as many as 38% of all murders of women are committed by intimate partners [5]. Although IPV is pervasive, there is substantial variation between and within countries with an estimated lifetime prevalence of IPV ranging from 20% in the Western Pacific to 33% in the African Region [5]. A recent study that included low- and middle-income countries in Sub-Saharan Africa (SSA) confirmed a wide variation in rates of physical or sexual violence also vary widely among adolescent women, ranging from less than 5% to more than 50%, with adolescents aged 15–19 years generally at higher risk than older women [6].

In SSA, the age of adolescence is a particular challenge for girls, who are doubly vulnerable because of their gender and lower social status as young people growing up in a patriarchal environment. Adolescent girls and young women in particular are exposed to violence by intimate partners, family members, and other persons. Violence affects the lives of these women beyond the event itself, with long-term consequences in the form of loss of self-esteem, lower educational attainment, lower income, anxiety, greater vulnerability to illness, social suffering, constant tension, depression, and suicide attempts [7, 8]. Lockdowns during the COVID-19 pandemic have also led to increased exposure of women to abusive partners.

Although violence is more common among female adolescents in SSA than among older women, many previous reviews focusing on the region either did not include young adolescents, did not present results specifically for this age group, or focused on adolescent sexual and reproductive health, while paying less attention to the experiences of violence among this age group. However, information about adolescents is important with regard to sustainable Development Goal 5 to achieve gender equality and empower all women and girls, [9–11], and especially for target 5.1 to eliminate all forms of violence against women and girls [5]. In particular, knowledge about the role of socio-economic status in IPV, and *vice versa* of IPV in socio-economic status, is important for designing interventions to end violence against adolescent women. This has become even more important in the pandemic era of COVID-19, as dramatic increases in IPV were observed during the COVID-19 lockdown. For example, in Kenya, Malawi, and Sudan [12], as well as other sub-Saharan African countries, COVID-19 led to an escalation of violence against women and girls. This was also exacerbated by the deteriorating economic situation of many households.

Socio-economic status (SES) includes not only the economic level but also educational attainment, financial security, and perception of social status and class. Outside SSA, socio-economic status (SES) is one of the most important factors influencing IPV among adolescent girls and young women, with higher educational attainment, higher socio-economic levels, and higher annual household income being associated with a lower likelihood of women being victims of violence [13, 14]. However, to date, there is no overview of the literature on the relationship between socio-economic status (SES) and IPV among adolescent girls and young women in SSA. Therefore, this review aims to capture the existing evidence on socio-economic factors influencing IPV among adolescent girls and young women aged 15–24 years in SSA. The findings of this review will contribute to the discourse on the complex relationship between IPV and SES among young women living in heterosexual relationships and provide a guide for policymakers and other stakeholders to curb the problem of IPV among young people and develop targeted interventions. This will help address the enormous problem of violence and the implementation of policies to achieve SDGs 3 and 5, which focus on gender equality and good health for all.

## Methods

A scoping review methodology was used to address the broad exploratory nature of the research question [15]. Online databases were used to identify studies published between 2015 and 2022. This scoping review was designed based on methodological guidance proposed by Peters and colleagues for JBI [16] with the use of population, concept, and context (PCC) to ensure key elements are reflected in the title for easy identification by the reader. The study also followed a framework provided by Arksey and O’Malley [15] for conducting a scoping review: (i) identifying the research questions and defining the eligibility criteria, (ii) identifying relevant studies by conducting an extensive search, (iii) making the study selection and appraising its quality, (iv) synthesizing the included studies (charting the data) and presenting the findings by using the Preferred Reporting Items for Systematic Reviews and Meta-Analysis (PRISMA) as an extension for the scoping review checklist [17]; (v) compiling, summarizing and reporting; (vi) consulting. Primary studies were appraised using the Mixed Methods Appraisal Tool, Version 2018 [18]. The protocol was registered in the Open Science Framework on 6th April 2022 with Identifier: DOI 10.17605/OSF.IO/VMFD2. The objectives of the current study are threefold: to identify how demographic characteristics influence the prevalence of IPV among AYW, the relationship between socio-economic factors and IPV among AYW, and how IPV affects the later socio-economic attainments of young women.

### Search Strategy (Identifying Relevant Studies)

Our goal was to synthesize the current evidence on how the socio-economic status of female adolescents and young women influences the experience of intimate partner violence. In this review, “intimate partner violence” is defined as “a pattern behavior within an intimate relationship that includes physical or sexual violent acts, often accompanied by emotional aggression and controlling behavior, enacted by a current or former intimate partner (i.e., spouse, boyfriend/girlfriend, dating partner, or ongoing sexual partner)” [19, 20]. We searched for peer-reviewed publications in English on IPV in SSA countries published between January 2015 and July 2022. A combination of keywords and medical subject heading (MeSH) terms was developed by the research team and reviewed with an expert medical librarian. A two-step search was carried out to ensure a complete search through online avenues. The following electronic databases: Medline, Embase, PsycINFO, CINAHL, Sociological Abstracts, Social Services Abstract, Web of Science, and African Index Medicus were used to identify published studies. All the articles retrieved were imported into the Covidence platform for a second analysis by screening selected relevant studies. Detailed search terms were already prepared and presented.

### Inclusion Criteria

The search was limited to a period of 7.5 years to capture current studies and the lessons learned during this time, that will help reshape advocacy efforts to address IPV among young women. The search included studies on the following topics: IPV, sexual violence, physical violence, emotional violence, economic violence, psychological violence, spousal violence, partner violence, dating partner violence, ongoing sexual partner relationships, and experience of IPV across the life course, under the condition that information on young women’s relationship empowerment, and economic empowerment of girls were simultaneously available. All study designs were included (qualitative, quantitative, and mixed methods studies) that reported original research and were published in a peer-reviewed journal. The search string is available as an online document.

### Exclusion Criteria

Studies of non-male violence against girls were excluded. Studies reporting different outcomes (e.g., mixed-gender reports) were also excluded, as well as all desk reviews, case reports, commentaries, book reviews, and editorial blog posts.

### Article Selection

In this review, comprehensive title and abstract screening were performed by searching and uploading all retrieved articles on the Covidence platform. Two reviewers independently screened titles and abstracts against the inclusion and exclusion criteria. The third reviewer helped to resolve any discrepancies in the final phase of the screening process, based on which the final decision on inclusion in the full-text review was made. In the second phase, the reviewers assessed the full text of the selected articles, and also checked the references in case there were other articles relevant to the study that could have been additionally searched. The PRISMA flowchart [17] was retrieved from the Covidence website to illustrate the process of screening articles and the inclusion and exclusion of articles.

### Data Extraction

The first two authors performed the data extraction for all selected articles. A spreadsheet was created for the extraction process (see table below), which was strictly followed. The extraction sheet listed the author’s aims of the study/objectives, study methods, and key findings related to the objectives of this review, tabulated in an Excel sheet for each publication. In addition, the year of publication, study population, geographic location, and conclusions/implications of the main findings were listed.

### Strategies for Data Synthesis

The articles included in the review reported qualitative and quantitative findings. Results were based on the objectives of the review. We provide a narrative synthesis of the findings from the included studies that focus on the prevalence of IPV among adolescents and young women by socioeconomic status, the relationship between socio-economic factors and IPV, the socio-economic challenges encountered by the study’s target populations, and the impact of IPV on adolescents and young women’s subsequent socio-economic attainments.

### Risk of Bias and Quality Assessment

Two review authors (OOI, NZ) independently assessed the risk of bias in the included studies by examining the appropriateness of the study objectives, design, method, participants recruited, data collection method, data analysis, and presentation of findings. A quality appraisal tool that focused on quantitative and qualitative methods, the Mixed Methods Appraisal Tool, Version 2018 [17], was used. Disagreements between the two review authors about the risk of bias in specific studies were resolved through discussion, with a third reviewer consulted as needed.

## Results

### Presentation of Results

**FIGURE 1:**
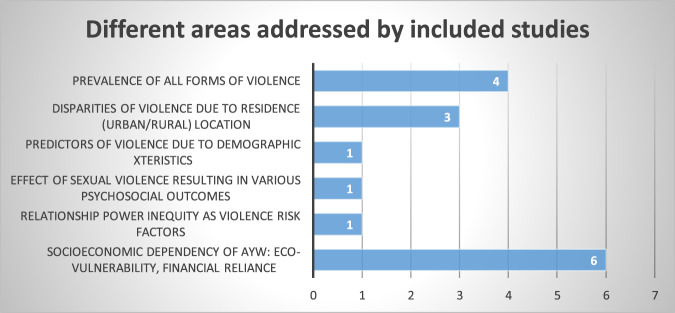
Bar chart showing key focus areas addressed by included studies (Sub-Saharan Africa, 2015–2022).

The extracted information was evaluated using descriptive statistics and thematic analysis. The final results were presented in tables and charts and organized into themes according to the objectives for in-depth discussion. The article information is presented in a table and includes the year of publication, sample size, the country where the data was collected, authors, the study design, study scope, and result and conclusion see Table 1. The narrative summary includes study details, key findings, and research gaps relevant to further studies, policy, and action. Also, specific areas addressed by the included studies are in the bar chart (Figure 1) below.

**TABLE 1:** Population, concept, and context (PCC) framework. Sub-Saharan Africa, 2015–2022.

Criteria	Determinant
Population	Adolescent and young women aged 10–25
Concept	Socio-economic factors associated with IPV (physical/sexual/emotional/psychological)
Context	Sub-Saharan African countries

### Screening Results

**FIGURE 2:**
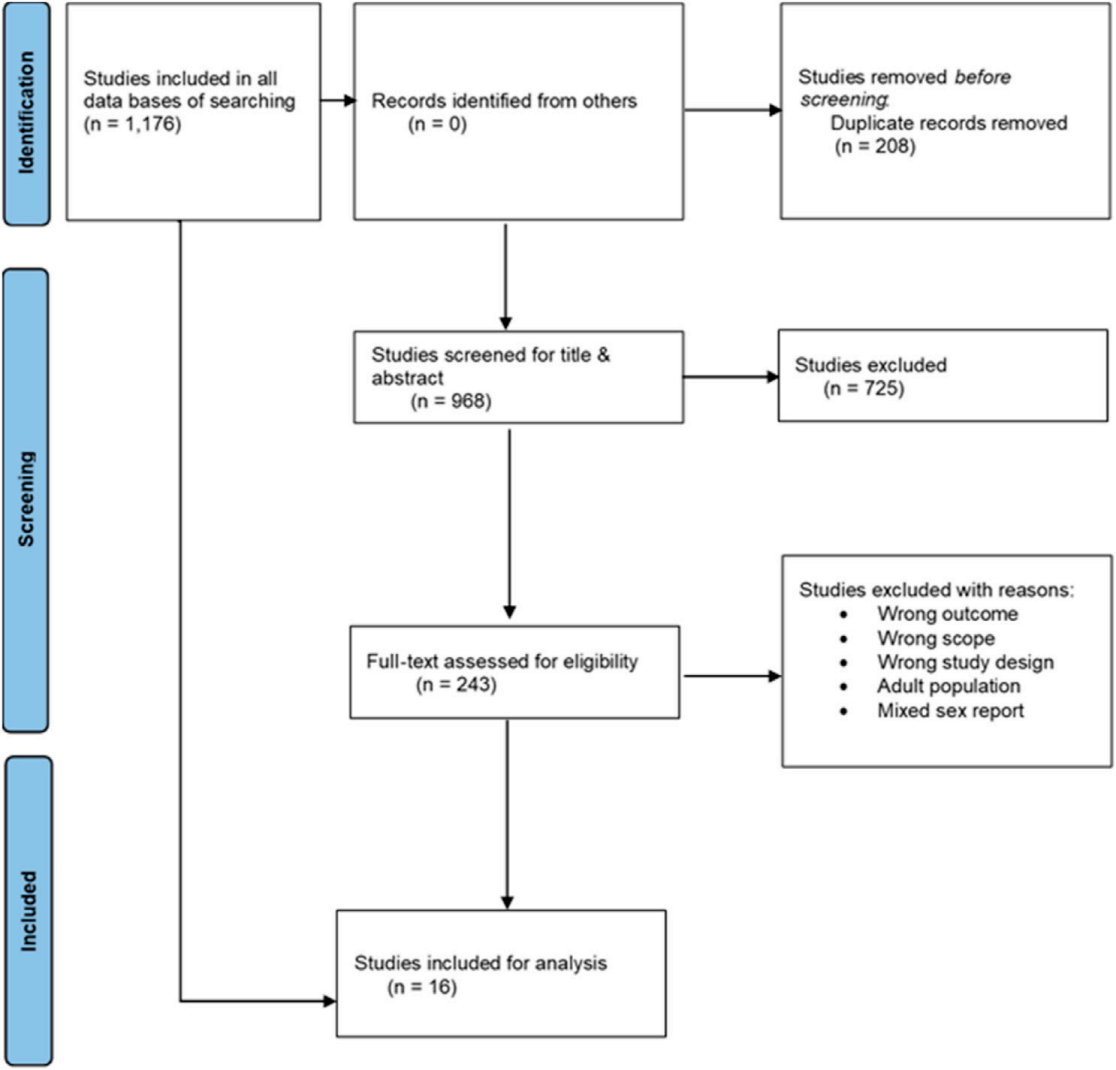
PRISMA flow diagram (Sub-Saharan Africa, 2015–2022).

Our search retrieved 1,176 articles. A total of 208 duplicates were removed, the titles and abstracts of 968 studies were screened, 50 studies were classified as irrelevant, and 918 studies were assessed for full-text eligibility to ensure we had captured all relevant studies. 902 studies were later excluded, leaving 16 articles for extraction from the Covidence platform (Figure 2).

### Characteristics of the Included Studies

All included studies were published in peer-reviewed journals [21–36], 10 reported quantitative findings from cross-sectional studies [23–28, 30–32, 34], 4 were qualitative [21, 34–36] and 2 had used mixed methods [22, 29]. Out of the 16 studies, 2 were conducted in Uganda [23, 33], 4 in Nigeria [21, 27, 35, 36], 1 in Ghana [34], 3 in South Africa [22, 28, 32], 1 in Mozambique [30], 4 in Kenya [24, 25, 29, 31] and 1 in 5 countries combined (Burkina Faso, Kenya, Malawi, Nigeria & Tanzania) [26].

All 16 studies explain socio-economic factors influencing IPV among adolescent girls and young women in SSA. Four studies focused on the prevalence of IPV experiences among adolescent girls and young women and described physical, sexual, psychological, emotional, and economic abuse. Three of the studies addressed disparities in experiences of violence related to adolescents’ residence. One study looked at predictors of violence, including age, education level, partner education level, alcohol use, and economic status. One study also addressed the unequal distribution of power in a relationship according to gender. Six studies more directly addressed the issue of socio-economic dependencies that made adolescent girls and young women vulnerable in their heterosexual relationships; economic vulnerability, lack of economic empowerment, lack of personal savings, and financial dependence on the partner increased the risk of experiencing violence.

### Prevalence, Nature, and Severity of IPV Among AYW by Socio-Economic Status

We found the prevalence of IPV among AYW in all of the 16 included studies in the 9 Sub-Saharan African countries, though it varied across countries. Four of the studies specifically addressed the experience of IPV among AYW in sub-Saharan African countries. One study from five countries (Burkina Faso, Kenya, Malawi, Nigeria, and Tanzania) [26] estimated the prevalence of teenage pregnancy and estimated the correlation with physical violence. The highest prevalence of physical violence was recorded in Nigeria among pregnant adolescents, who were five times more likely than non-pregnant to be affected, due to the socio-economic status of AYW. Also, a study from Kenya reported the severity of IPV by socioeconomic status among adolescent girls and young women (AYW) which were; psychological 33.1%, physical 22.9%, and sexual 15.8% [25]. This study shows that AYW who were previously married or living with a partner or slept hungry at night were at higher risk for psychological violence. It also showed that adolescents aged 10–14 years who were in wage-earning jobs or whose families had no money were at higher risk for violence. The study showed further that young women who had sex due to food insecurity are at higher risk for sexual violence. Young women whose partners were unemployed were almost twice as likely to be victims of IPV [30].

### Socio-Economic Risk Factors of IPV Among Adolescent Girls and Young Women in a Heterosexual Relationship

Socio-economic factors affecting young women’s IPV experience were discussed in two ways: (1) socio-demographic household characteristics as risk factors for IPV, and (2) young women’s financial dependence as a risk factor for IPV.

### Socio-Demographic Household Characteristics as Risk Factors for IPV Among AYW in Heterosexual Relationships

Seven studies associated several socio-demographic characteristics with IPV among adolescent women. The studies reported risk factors predicting IPV experience among AYW: age, region, living in rural or low-income communities in urban areas, age at marriage, education level, wealth status, and number of living children, spouse’s education, jealousy, and alcohol consumption were reported to be significantly affecting IPV experience [27, 28]. A study from Kenya also found that lower socioeconomic status and living in rural areas, being associated with poverty and women’s low empowerment, was strongly associated with an increased likelihood of IPV and also negatively affected reproductive healthcare utilization [31]. In a study from Mozambique, partners’ unemployment status was found to be a risk factor for experiencing IPV: Partner unemployment implies more opportunities for abuse of AYW at the slightest perceived “mistakes” [30]. This was especially problematic for women who could not rely on social support to control their husbands when they were not actively part of a religious community.

Nonetheless, a previous study from Nigeria by Antai [37] found that also women with higher socioeconomic status in terms of higher income compared to their partners were more likely to suffer IPV. This contradictory finding suggests that the socio-economic independence of AYW does not necessarily reduce the experience of IPV, especially in patriarchal systems which are still prominent in SSA. However, a study from South Africa reported that young women in urban areas who experienced IPV and had a higher socio-economic status were better able to manage their psychological distress compared to rural residents [28]. Generally, few of the studies reviewed reported differences in psychosocial wellbeing.

Marital status also affects the risk of IPV. A study from Kenya reported that adolescent girls and young women who were previously married or living with a partner were at risk of IPV experiences [25]. The study investigated how adolescent girls and young women’s experiences of violence varied by place of residence. Insignificant disparities in IPV experience existed between urban and rural areas. However, there were absolute disparities in the experience of sexual violence between ever-married young women living in rural and urban areas, and psychosocial outcomes of IPV varied by marital status among young women. The authors attributed differences to residence-related structural and socio-cultural differences between areas.

A study from Uganda however found that the ever-married and never-married young women were both affected by IPV [23]. Those who have been married before and are now divorced or living alone, and those who are not yet married but are living with an intimate partner, were more often affected by IPV.

### Financial Dependency of AYW Influenced IPV, Eco-Vulnerability, and Financial Reliance

Six studies reported on how financial vulnerability contributes to adolescent AYW experiences of violence in SSA due to their economic vulnerability, financial reliance, and lack of savings. A study in Nigeria found that economic vulnerability limits girls’ financial independence, which in turn increases their financial dependence on their male partners, thereby increasing their risk of IPV [21]. Financial dependence makes it difficult to leave an abusive relationship, especially when a young woman is pregnant or has a child [36, 38], it poses a long-term threat to their financial stability. Women who were married as children in Ghana also reported being economically dependent on their partners, which led to male autonomy in their households and increased the risk of experiencing all forms of IPV [34]. In informal settlements in Nigeria as well, financial difficulties triggered IPV, and economic constraints were one of the reasons cited by adolescent girls and young women for engaging in transactional and commercial sex despite the heightened risk of violence from clients [35]. However, a study in Kenya showed that having savings by AYW can be protective against IPV, though economic empowerment alone does not reduce the risk of IPV [29].

AYW’s food insecurity was associated with an especially high risk of experiencing IPV: Transactional sex, associated with food insecurity and with survival strategies, increases the risk of sexual violence against adolescent girls and young women [25, 35].

### Effect of IPV on Later Socio-Economic Attainments of AYW in SSA

Most articles reviewed did not explicitly discuss the impact of IPV on young women’s later socio-economic levels, beyond that it was generally believed that better socio-economic conditions are likely to reflect better living conditions and that less stress related to financial problems may reduce the occurrence of violent behaviors by live-in partners. Although the experience of violence during adolescence affects all aspects of job preparation, skills, earnings, and occupational attainment, only one article explicitly mentioned that even though all socio-economic categories are affected by IPV, young women from lower SES households are more likely to be exposed to various forms of IPV that in turn affect the level of later SES attainment [39].

## Discussion

This review aimed to synthesize evidence for socio-economic factors influencing IPV among AYW in SSA. The review covered studies conducted between 2015 and 2022, which aligns with the start of the Sustainable Development Goals (SDGs) [9, 10], including all papers addressing physical, emotional, economic, psychological, and sexual violence perpetrated by a male intimate partner against a female partner aged 15–24 years. Although one systematic review measured the direct costs of violence against girls and women in terms of healthcare costs and loss of productivity [40] in SSA countries, data on the consequences of IPV on young women’s socio-economic attainment was limited.

The review found 16 studies that focused directly or indirectly on how adolescent women’s socio-economic status affects their experiences of IPV. Most studies in our review examined the impact of AYW’s socio-economic status and its overall effect on IPV and its consequences. In contrast, no study explicitly examined the effects of IPV in adolescence on later socio-economic attainment.

IPV is recognized worldwide as a public health problem and young women are at greater risk of IPV [41]. The results of this study confirm the importance of socio-economic factors influencing IPV among young women in SSA. The findings suggest that the extent of IPV in relation to young women’s socio-economic status is a global problem that is even more pronounced in SSA achieving the laudable SDGs 3 and 5 appear challenging. Women’s economic empowerment not only benefits the development of society as a whole but also enables women to decide for themselves how best to organize their lives in an equal relationship. Women’s rights, including the right to health, require the empowerment of girls in terms of education and economic participation, as well as the involvement of men to prevent the recurrence of violence against girls and women [42]. Efforts in SSA are not yet sufficient to reduce IPV experience among AYW and to improve their health and socio-economic status. Unequal power relations stemming from social norms that disadvantage AYW continue to lead to violence against women, endangering the health of young girls, and imposing “high social and economic costs on communities and society as a whole” [42].

Young women who have no financial means are exposed to all forms of violence. Economic vulnerability increases a girl’s financial dependence on her male partner and thus the risk of violence [21]. AYW with limited or no income are financially dependent on their partners. This economic dependency contributes significantly to IPV in SSA. Low socio-economic status, low educational attainment, economic deprivation, and inability to meet daily needs thus predict the experience of IPV [8, 40], while women are unable to leave the violent relationship due to their financial dependence on their partner, which poses a long-term threat to their employment or financial stability [43, 44].

It has further been shown that low social support increases the risk of IPV [45, 46]. In India for example, it was found that socio-economic status alone does not influence the experience of violence, but that a low social support system increases the experience of violence [46]. Many of the articles we screened focused on individual social support rather than on the structural, socio-economic factors as a determinant of IPV. We believe however that improving the economic status of AYW must be prioritized, for example by improving the educational attainment of young girls and by creating employment opportunities, prioritizing jobs without gendered wage inequalities. This will inevitably increase the socio-economic status of AYW in SSA, and thus lower their vulnerability to IPV.

Age, level of education, place of residence, number of living children, marital status, employment status, and income were the most common single demographic variables mentioned to impact IPV. However, the strength of these associations was not reported in most studies, as they were discussed as common determinants of different forms of violence experiences. For example, several studies mentioned that age influences IPV. This finding is consistent with an earlier study conducted in low- to middle-income countries (Zimbabwe, Cameroon, Congo, Namibia, Senegal, Sierra Leone, Zambia, and Rwanda) in which adolescents aged 15–19 years were at higher risk of IPV compared to older women [6]. This confirms that especially adolescent girls have limited power to leave a relationship, lack social support, may be threatened, be financially unable to leave an abusive relationship, may not report IPV to the authorities, and will thus remain in the abusive relationship.

The marital status of young women has also been found to be a risk factor for violence against women. A woman who is or was previously married was more often affected by violence against women than a woman who is single but in an intimate relationship. A longer exposure may explain the higher prevalence of violence among married, widowed, and formerly married women or women with a child, compared to single women with an intimate partner [25, 47].

Furthermore, some of the results are seemingly contradicting. For example, low socio-economic status was generally found to increase the risk of IPV. However, the employment status and income of either partner determines the risk of IPV differentially: in a study from Nigeria in 2011 the authors found that violence and abuse increased against young women when they had a higher income compared to their partner or husband who was also employed [37]. It should be considered that there may be three different explanations linking SES and IPV: 1) Poverty and economic dependency increase the risk of IPV, 2) Higher levels of education and socio-economic status of the AYW are associated with a higher likelihood of reporting violence (as violence may be normalized in impoverished settings), 3) Higher education and socio-economic status of the AYW may challenge the superior status of men in patriarchal societies and thus trigger violence. In addition, AYW with a higher level of education, and therefore a later start to earning, may be economically disadvantaged compared to women in trade. The claim to encourage AYW to further their education or acquire vocational skills to enhance their financial independence and socio-economic status remains important, but eventual trade-offs of women’s economic empowerment must be recognized and addressed.

The prevalence of IPV in SSA varies from country to country and region to region [26]. This was consistent with previous reviews [3, 48]. Previous studies have also already found that violence against young women was related to socio-economic status [49–51]. This suggests that IPV is a consequence of persisting poverty, a patriarchal system, and the societal tolerance of IPV continues to be widespread [30, 52]. It must be considered though that there is some epistemic inequality in research between contexts, as common research questions vary between high- and low-income countries. While the role of poverty has been explored as a determinant in all contexts, the effect of IPV on women’s income has mainly been investigated in high-income countries. In high-income countries it has long been shown that women who have experienced violence have difficulty finding stable employment or increasing their income [53]. However although the reviewed studies emphasized the possible impact of IPV on the socio-economic status of young adults in all areas of life our study was not able to determine the impact of IPV on the socio-economic attainments of young adults because no study explicitly examined this relationship. Nevertheless, several studies mentioned financial instability due to IPV experiences, job insecurity among young adults due to IPV, and lack of savings, which makes young adults more vulnerable [21, 29, 30].

We conclude that measures to reduce violence against young women should be targeted based on the circumstances and configuration of each country concerning regional disparities. Young women who are most at risk of IPV are likely to be out of school, attending school, have a low socio-economic background or are unemployed. Education has always been the most important single measure to reduce exposure to violence. Information about the impact of violence against AYW on mental health, the harmful effects of psychological distress, and the unimaginable situation young women can find themselves as sex workers due to their low socio-economic status is particularly important at all levels of society. In addition, AYW needs to be educated about support structures to increase their decision-making power, and how they can best get help given the experiences they have had in their relationships. Social support is very important for young women experiencing violence, but the government should increase formal support structures if an end or drastic reduction in the experience of violence is to be achieved. Currently, AYW has support primarily from their friends, family, and religious institutions, as IPV has been seen in the past as a family affair, neglecting the structural causes of violence. But over the years also informal support systems have been weakened in ways that have nurtured an increase in IPV in SSA.

This review contributed to the body of knowledge by examining the socio-economic dependence of female youth on their partners and how this influences IPV experiences alongside eco-vulnerability and financial dependence. It also examined the socio-demographic characteristics that serve as risk factors for re-victimization, namely, age and marital status. We also examined the prevalence of all forms of violence, from physical, emotional, economic, sexual, and psychological violence to differences in place of residence and psychosocial outcomes. Secondly, we found no study that explored how IPV experience may influence women’s socio-economic attainment in sub-Saharan Africa. This is a major research gap that should be explored. The Sustainable Development Goal (SDG) 1, “No Poverty” is focused on ending poverty in all forms by 2030. Therefore, there is a need to guarantee basic income or unconditional cash transfer to encourage AYW to stay in school, and educational attainment threshold for young women.

### Strength and Limitations

This study is a unique review that addresses the relationship between socio-economic factors and IPV among young women in SSA. The review methodology was rigorous. The screening process involved three independent reviewers who were aware of the study’s objectives and eligibility criteria. In cases where there were conflicts in the selection decision, the three came together to resolve them. The use of the Covidence online platform for screening was also beneficial and contributed to accurate and detailed screening. Our study focused on socio-economic status as a determinant of the occurrence of violence and the type of violence, as well as a factor influencing the impact of violence on children in adolescence.

However, despite the strengths noted above, this review is subject to some limitations. First, none of the studies we reviewed reported the impact of IPV on later socio-economic attainment; these were found only in papers from developed countries. Second, only studies written in English were included, due to our limited timeframe we could not access studies from other languages due to time to translate into English. There is a possibility that we excluded some relevant articles that would have been useful for this review. Overall, we identified a need for more long-term research on the origins, facilitators and consequences of IPV, and how these are intertwined, in order to inform gender-based violence prevention and response programs.

### Conclusion

Our results demonstrated that the socio-economic vulnerability of adolescent females overrides all other risk factors that contribute to the experience of violence. It is important to note that no study examined the impacts of IPV in adolescence on later socio-economic attainment. This is one of the critical gaps our study found, future research should look into how IPV affects the long-term socio-economic attainment of young women. Understanding that a young woman is not only able to earn her own income but also has the right to her savings will reduce her vulnerability to IPV and the risk of re-victimization. Therefore, an effective intervention for young women is to continue empowerment programs related to access to schooling for girls, entrepreneurship, and education on personal savings, as some women do not have a personal account, which reduces the chances of having their savings. In addition, interventions also need to address men, educating them on women’s rights. Government support for victims/survivors of violence, alongside fighting impunity among law enforcement agents on violence against women, is necessary to enable young women to leave violent relationships. Therefore, relevant policy gaps addressing these issues are essential to combat IPV in SSA.
