# Supplementary material for: Socio-Economic Factors Influencing Intimate Partner Violence Among Adolescents and Young Women in Sub-Saharan Africa: A Scoping Review
Source: Public Health Rev. 2025 Jan 3;45:1607041. doi: 10.3389/phrs.2024.1607041 (PMC11738612; doi:10.3389/phrs.2024.1607041)
Supplement: Supplementary file 1 [file Table1.docx]

**Appendix 1**

**Table 1 Summary of research findings from included source publications (n 10)**

| Author/Date | Settings/Country | Study aim | Study design | Study participants | Findings | Conclusion/Implication |
| --- | --- | --- | --- | --- | --- | --- |
| Fawole *et al*  [1] | Ibadan, Nigeria | To explore the motivations for and relationship dynamics that may promote girls’ vulnerability for intimate partner violence among girls residing in low-income communities in Ibadan. Nigeria. | Qualitative study | 122 girls (age 15-19) (14 FGDs) | Girls reported that leaving an abusive relationship was especially difficult when girls rely financially, were sexually involved, pregnant, or have had a child with the male partner. Friends/peers, were often the source of support for intimate partner violence. | Secrecy of girls’ relationships with male partners furthers girls’ risk for intimate partner violence as well as the negative consequences. Peer-based models may be most effective, given that girls tend to seek relationship related support from peers, rather than from their parents. |
| Ikuteyijo *et al [2]* | Nigeria | To explore the lived experiences of violence and health related harm among vulnerable young female sex workers in urban slums in Ibadan and Lagos, Southwest Nigeria. | Qualitative study | 12 female (15-24) (IDI) | The major motivation for engaging in commercial sex work was for economic reasons. However, there were inherent risks involved particularly for the vulnerable young people. | Exposure to violence further exacerbates the plight of younger girls who are involved in commercial sex. Rather than criminalizing this vulnerable group, there is need for proper regulation and protection for them. |
| Amoah *et al [3]* | Ghana | To describe Ghanaian women who married as children to examine their IPV experiences, and the factors which according to these women, contributed to these experiences. | Qualitative study | 15 women (who married before age 18) | Most of the participants had experienced physical, emotional, sexual, and economic abuse from their intimate partners. Respondents also indicated they were economically dependent on their partners and/or had no or only little autonomy in their households. Polygyny and other cultural beliefs and practices were identified as contributing factors to IPV in their relationships. | Better education would play a significant role in curbing early or child marriage. It is recommended that the Government of Ghana prioritize female education. It is important to recommend incentives to ensure the barriers to education are eliminated. |
| Amone-P'Olak *et al [4]* | Uganda | To investigate the effects of sexual violence on the odds of different psychosocial outcomes (depression, psychotic symptoms, somatic complaints, conduct problems, daily functioning, community relations, and stigma) among formerly abducted girls in Uganda. | Cross-sectional study | 210 girls, age (18-25) | Up to 65 % of formerly abducted girls reported experiencing sexual violence while in rebel captivity | These findings suggest that exposure to different levels of sexual violence may not have the same effects on various dimensions of psychosocial outcomes. |
| Ajayi *et al [5]* | South Africa | To determine the prevalence and associated factors of sexual violence among adolescent girls and young women (AYW) in one of the economically disadvantaged universities in South Africa. | Cross- sectional study | 451 adolescent and young women age (17-24) | Several factors are responsible for insufficient financial support, alcohol use, and many of the perpetrators were close acquaintances, which makes reporting difficult. | Alcohol use and inadequate family financial support were associated with exposure to sexual violence. Alcohol use reduction and conditional cash transfer programs for indigent AYW are potentially relevant interventions. |
| Burns *et al [6]* | Kenya | To examine the association between IPV (physical and sexual abuse) and direct and indirect maternal care-seeking behavior, within the context of high rates of under-five mortality. | Cross-sectional study | 1406, Ever-married women age (15-24) | Women who had experienced IPV were less likely to complete a minimum 4 antenatal visits. Lower socio-economic status and living in a rural area were strongly associated with increased likelihood of IPV. | IPV as it relates to women’s empowerment, poverty, and maternal and child health policy and programs (SDG goals 1, 3, and 5). The study highlights three priority strategies to complete the unfinished agenda of the MDGs: informing policy, developing prevention interventions, and future research. |
| Maguele *et al [7]* | Mozambique | To determine the prevalence and the factors associated with intimate partner violence in young women (aged 15–24 years) attending secondary schools in Maputo, Mozambique. | Cross-sectional study | 431 Young women age (15-24) | The risk of IPV was associated with young women lacking religious commitment, and if the partner was unemployed. Further results showed odds of being abused remained higher, among young women if the partner was employed, and among the young women who believe males are superior to females | Need to improve social interactions that promote violence free relationships, gender egalitarian norms, and physical and emotional wellbeing for young women. Comprehensive programs should incorporate socio-economic empowerment strategies to increase women’s autonomy. |
| Muthengi *et al [8]* | Kenya | To examine the association between work and experience of physical violence among married adolescents, and to understand the impact of access to independent financial resources on this risk. | Mixed methods | Married girls of 452 quantitative and 32 for qualitative study | While economic empowerment in the form of work for married adolescent girls can be associated with increased risk of experiencing violence, having savings can be protective. Financial difficulties faced by families living in informal settlements, and financial conflicts can be triggers of violence in marriages. | Women’s management of and access to financial resources can potentially help to reduce the risk of IPV. |
| Manyema *et al [9]* | South Africa | To investigate the associations between interpersonal violence and psychological distress (PD) among rural and urban young women. | cross-sectional study | 926 non-pregnant young women aged (18-22) | Higher SES seemed to protect against PD in the urban residents but had no apparent effect on the rural residents. Young women residing in the rural area were less likely to report PD compared to the urban young women. | Experiences of interpersonal violence are higher in urban than in rural young women and they do not seem to be associated with PD in the latter. Interventions for violence and mental health in the urban areas may therefore not necessarily translate into the same gains in rural areas |
| Onipede [10] | Nigeria | To examine the predictors and implications of intimate partner violence (IPV) against married female youths. | Cross-sectional study | 4574 married female youths aged (15-24) | Significant predictors of IPV in this study include age, region, age at first marriage, education, wealth status, number of living children, spouse’s education, jealousy, and alcohol consumption. | To lessen IPV among married female youths, policies to discourage early marriage, promote post primary schooling, and discourse on age alcohol consumption are imperative. |
| Tetteh *et al [11]* | Burkina Faso, Kenya, Malawi, Nigeria & Tanzania | To estimate the prevalence of Teenage Pregnancy (TP) and Physical Violence (PV) and further assesse the relationship between TP and PV in five Low-and-Middle-Income Countries (LMICs). | Cross-sectional study | 26,055 teenagers aged (15-19) for all five countries | The highest prevalence of PV was recorded among Nigerian adolescent women compared to other countries. The prevalence of PV among adolescent women who were pregnant was approximately 5-folds significant compared to those who were not pregnant. | Counter-intuitively, pregnant teenage women were at a higher disadvantage to ever experience physical violence. Intervention should target PV and TP by adopting a gender-sensitive approach to eliminate physical violence, particularly among teenagers to prevent TP. |
| Orindi *et al* [12] | Kenya | To estimate the prevalence, severity and identify predictors of violence among adolescent girls and young women (AYW) in informal settlement areas of Nairobi, Kenya, | Cross-sectional study | 1081 adolescent girls and young women aged (10-22) | Among 1081 AYW aged 15–22 years, psychological violence was the most prevalent in the past year (33.1%), followed by physical violence (22.9%), and sexual violence (15.8%). AYW who were being previously married or lived with a partner, or were sleeping hungry at night during the past 4 weeks had greater odds of experiencing psychological violence. Engaging in sex and food insecurity increased chances for sexual violence. | The violence is intimately related to some of the social as well as cultural norms but, seem to be driven by the economic circumstances under which these girls find themselves in. The need for increased effort geared towards addressing drivers of violence as an early entry point of HIV prevention effort among the vulnerable group. |
| Pulerwitz *et al [13]* | Kenya | To test the Sexual Relationship Power Scale (SRPS) among adolescent girls and young women (AYW) and examine associations with select health outcomes. | Cross-sectional study | 1101 adolescent girls and young women aged (15-24) | Most respondents reported limited power in their sexual relationships, however older respondents consistently reported lower levels of power. | Low relationship power was a consistent predictor of partner violence, as well as an important predictor of HIV risk. Common implementation strategies focus on targeted approaches or comprehensive approaches and creating an enabling environment for AYW empowerment |
| Mutumba *et al* [14] | Uganda | To identify which groups of young women are most vulnerable to sexual violence (SV) and characterize the magnitude of these disparities to inform intervention efforts and resource allocation to eliminate SV in Uganda. | Cross-sectional study | 1894 of ever-married and never-married young women aged (15-24) | For all young women combined, the results indicated small or insignificant inequalities in SV between urban and rural residents but there were large absolute and relative inequalities in SV between ever-married women residing in urban and rural areas. These place-based inequities in SV could be attributed to the structural and socio-cultural features of these environments. | The findings highlight profound place-based inequities in SV (urban/rural and region) among ever-married women. Therefore suggests that SV prevention programs should be regionally targeted to address the regional inequities in young women’s risk for SV by utilizing a multi-sectoral approach aimed at strengthening young women’s education attainment, reducing household poverty. |
| Zembe *et al* [15] | South Africa | To assess the extent and correlates of intimate partner violence (IPV), explore relationship power inequity and the role of sexual and social risk factors in the production of violence among young women reporting more than one partner in the past three months | Mixed methods | 259 young women aged for quantitative (16-24) and 36 for qualitative | There are several factors that make IPV socially accepted to victims, perpetrators and onlookers; however the critical role played by socio-cultural norms that encourage hierarchical sexual relationships, and valorize aggressive masculinities while promoting timid, subservient performances of femininity | The social acceptance of violence and community bystander apathy in the study community, calls for interventions that emphasize the involvement of community members as allies in efforts to combat gender based violence |
| Salawu *et al* [16] | Nigeria | To explore the experience of economic vulnerability and its effect on girls’ future aspirations, relationships, and financial reliance on male partners, as well as risk for intimate partner violence (IPV) and related health consequences. | Qualitative study | 122 female aged (15-19) | The findings showed that economic dependence on male partnerships increases girls’ vulnerability for IPV and related health effects. Participants reported that financial dependence on male partners makes it difficult to leave unhealthy relationships. | Economic vulnerability decreases girls’ financial independence, and in turn, increases girls’ financial dependence on male partners, risk for IPV, and risk for poor sexual and reproductive health. Interventions to support girls to achieve financial independence may help alleviate risks associated with economic vulnerability experienced by young girls. |

1. Fawole, O.I., et al., *Relationship dynamics with male partners among girls in low-income communities of Ibadan, Nigeria: Risk for violence and health related consequences.* J Adolesc, 2021. **87**: p. 74-85.

2. Ikuteyijo, O.O., A.I. Akinyemi, and S. Merten, *Exposure to job-related violence among young female sex workers in urban slums of Southwest Nigeria.* BMC Public Health, 2022. **22**(1): p. 1021.

3. Amoah, H.A., E.Y. Tenkorang, and P. Dold, *Experiences of intimate partner violence against women Who married as child brides in Ghana.* Journal of Family Violence, 2021. **36**(4): p. 455-466.

4. Amone-P'Olak, K., E. Ovuga, and P.B. Jones, *The effects of sexual violence on psychosocial outcomes in formerly abducted girls in Northern Uganda: the WAYS study.* BMC Psychol, 2015. **3**: p. 46.

5. Ajayi, A.I., E. Mudefi, and E.O. Owolabi, *Prevalence and correlates of sexual violence among adolescent girls and young women: findings from a cross-sectional study in a South African university.* BMC Womens Health, 2021. **21**(1): p. 299.

6. Burns, P.A., et al., *Intimate Partner Violence, Poverty, and Maternal Health Care-Seeking Among Young Women in Kenya: a Cross-Sectional Analysis Informing the New Sustainable Development Goals.* Glob Soc Welf, 2020. **7**(1): p. 1-13.

7. Maguele, M.S., et al., *Risk factors associated with high prevalence of intimate partner violence amongst school-going young women (aged 15-24years) in Maputo, Mozambique.* PLoS One, 2020. **15**(12): p. e0243304.

8. Muthengi, E., T. Gitau, and K. Austrian, *Is Working Risky or Protective for Married Adolescent Girls in Urban Slums in Kenya? Understanding the Association between Working Status, Savings and Intimate-Partner Violence.* PLoS One, 2016. **11**(5): p. e0155988.

9. Manyema, M., et al., *The associations between interpersonal violence and psychological distress among rural and urban young women in South Africa.* Health Place, 2018. **51**: p. 97-106.

10. Wusu, O., *Predictors and implications of intimate partner violence against married female youths in Nigeria.* Journal of Family Violence, 2015. **30**(1): p. 63-74.

11. Tetteh, J., et al., *Teenage pregnancy and experience of physical violence among women aged 15-19 years in five African countries: Analysis of complex survey data.* PLoS One, 2020. **15**(10): p. e0241348.

12. Orindi, B.O., et al., *Experiences of violence among adolescent girls and young women in Nairobi’s informal settlements prior to scale-up of the DREAMS Partnership: Prevalence, severity and predictors.* PLoS One, 2020. **15**(4): p. e0231737.

13. Pulerwitz, J., S. Mathur, and D. Woznica, *How empowered are girls/young women in their sexual relationships? Relationship power, HIV risk, and partner violence in Kenya.* PLoS One, 2018. **13**(7): p. e0199733.

14. Mutumba, M., S. Bhattacharya, and F.M. Ssewamala, *Assessing the social patterning and magnitude of inequalities in sexual violence among young women in Uganda: Findings from 2016 demographic and health survey.* Glob Public Health, 2022: p. 1-15.

15. Zembe, Y.Z., et al., *Intimate Partner Violence, Relationship Power Inequity and the Role of Sexual and Social Risk Factors in the Production of Violence among Young Women Who Have Multiple Sexual Partners in a Peri-Urban Setting in South Africa.* PLoS One, 2015. **10**(11): p. e0139430.

16. Salawu, M.M., et al., *Money, Power, and Relationships: Economic Vulnerability in Girls' Lives and Risk for Partner Violence Among Girls in Ibadan, Nigeria.* Violence Against Women, 2021: p. 10778012211058223.
